# Including outcomes other than cesarean section: An innovative perspective on Robson Classification

**DOI:** 10.1111/aogs.14370

**Published:** 2022-04-25

**Authors:** José Paulo de Siqueira Guida, Maria Laura Costa

**Affiliations:** ^1^ Department of Obstetrics and Gynecology University of Campinas Campinas Brazil

*Sir*,

We carefully read the article by Savchenko et al,^1^ presenting an innovative perspective on the application of the Robson Ten Group Classification. As far as we know, this was the first study to consider other outcomes besides cesarean section using this classification system. According to the Robson Classification,^2^ women admitted for childbirth are classified into one of 10 groups, which are totally inclusive and mutually exclusive. It provides data regarding the obstetric characteristics of the population analyzed and allows comparisons between different populations and hospitals. It also helps to evaluate the impact of policies to reduce cesarean section rates.

In their innovative analysis, Savchenko et al classified each delivery of a large national cohort according to Robson's Classification, and then evaluated the occurrence of several obstetric outcomes: operative vaginal delivery (OVD), obstetrical anal sphincter injury (OASIS), postpartum hemorrhage (PPH), and low Apgar index. They showed that, although total OVD and OASIS rates were low, among primiparous women those events were more frequent, whereas PPH was more frequent in groups where cesarean section rates were higher. Their study demonstrated that other adverse outcomes are also better understood when we know in which group they are more prevalent, allowing us to implement measures to reduce their occurrence and impact.

We previously showed that cesarean section rates are higher among women with preeclampsia.^3^ Considering the idea of Savchenko et al, we reanalyzed our database of 3102 women who delivered in a Brazilian maternity hospital from January 2017 to February 2018, considering as outcome the occurrence of preeclampsia. We observed a preeclampsia rate of 8.3%, ranging from 0 (group 9) to 26.5% (group 10). Groups 1 to 4 corresponded to 55.2% of our sample, contributing to 27.5% of all preeclampsia cases, and 46.5% of those cases were in group 10, which corresponded to only 14.6% of our sample. Group 5 was the largest group, comprising 23.4% of the population, with a preeclampsia rate of 6.2% and contributing to 17.4% of all preeclampsia cases.

The analysis allowed us to understand that the majority of our population could be considered to be at low risk of obstetric complications (groups 1 to 4); however, these groups contributed significantly to the overall preeclampsia rate. Therefore, even low‐risk women should be actively surveilled for blood pressure and other signs of preeclampsia. The increase in the cesarean section rate in Brazil in recent years explains group 5 as currently the largest group, emphasizing the urgent need to avoid first cesarean section among Brazilian women. Preterm preeclampsia is extremely high in our population, highlighting the impact of medically indicated preterm birth due to hypertensive pregnancy complications. Nevertheless, this finding also supports the need for better implementation of preventive interventions, such as low‐dose aspirin and calcium supplementation to women at high risk for preeclampsia.

We strongly suggest that other researchers who are currently studying the Robson Classification should consider Savchenko et al's proposal in their approach to data analyses, and we agree it could be useful for analyzing other outcomes rather than only cesarean section rates.
